# Malignancy-Associated Secondary EGJOO (Pseudoachalasia) Due to Infiltrative Gastric Adenocarcinoma

**DOI:** 10.3390/diagnostics16213458

**Published:** 2026-10-23

**Authors:** Jiaqi Yao, Peihong Li, Tao Guo, Xiaoqing Li

**Affiliations:** Department of Gastroenterology, Peking Union Medical College Hospital, Chinese Academy of Medical Sciences and Peking Union Medical College, Beijing 100730, China

**Keywords:** pseudoachalasia, primary achalasia, esophagogastric junction outflow obstruction, gastric adenocarcinoma, endoscopic ultrasound

## Abstract

Pseudoachalasia is an uncommon condition that clinically, radiographically, and manometrically mimics primary achalasia and is most frequently caused by a malignancy involving the gastroesophageal junction or gastric cardia. Distinguishing pseudoachalasia from a primary motility disorder remains challenging because conventional investigations may initially appear reassuring. We present a patient with progressive dysphagia and weight loss whose initial EGD was interpreted as showing no definite mucosal abnormality and whose HREM demonstrated a manometric pattern consistent with EGJOO. Progressive symptoms prompted repeat endoscopy and EUS; these revealed distinct abnormalities of the proximal gastric wall; targeted biopsies confirmed poorly differentiated gastric adenocarcinoma. Although the initial endoscopic examination showed no evident abnormality, HREM cannot visualize or histologically characterize tissue; therefore, it cannot exclude a structural or malignant etiology. This case illustrates that progressive symptoms should prompt re-evaluation.

**Figure 1:**
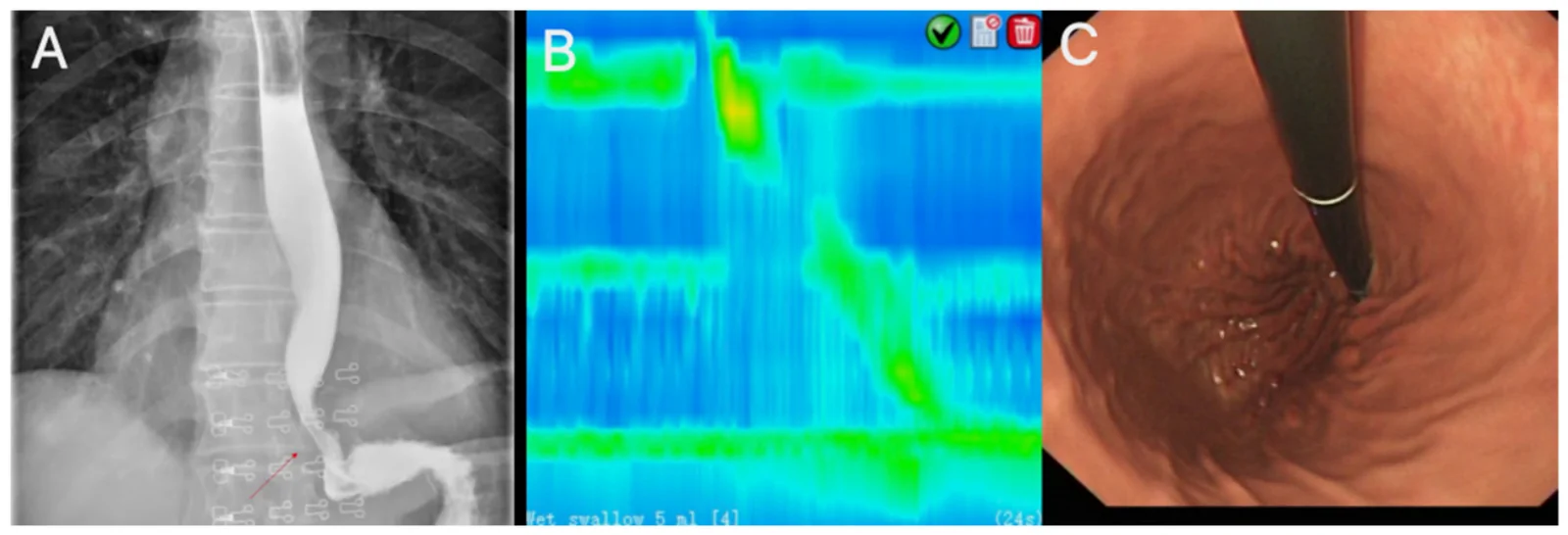
The initial evaluation for suspected achalasia was performed at an outside hospital. A 58-year-old woman was referred to our institution in July 2025 with a one-year history of progressively worsening dysphagia to both solids and liquids, beginning in July 2024 and accompanied by regurgitation and unintentional weight loss of 8 kg. She had no history of gastrointestinal disease, prior abdominal surgery, or malignancy. Physical examination was unremarkable. Routine laboratory investigations, including serum tumor markers, were within normal limits. Barium esophagography demonstrated distal esophageal narrowing with a bird-beak appearance (**A**), suggestive of achalasia. Achalasia is a rare primary esophageal motility disorder characterized by impaired lower esophageal sphincter relaxation and absent peristalsis, leading to functional outflow obstruction at the esophagogastric junction (EGJ) [1]. Its cardinal symptoms include dysphagia, regurgitation, chest pain, and weight loss. Diagnosis is typically based on high-resolution esophageal manometry (HREM), endoscopy, and barium esophagography [2]. HREM was subsequently performed. The median integrated relaxation pressures (IRPs) in the supine and upright positions were 22.36 and 17.42 mmHg, respectively, and increased intrabolus pressure was observed in 30% of supine wet swallows with preserved peristalsis (**B**). According to Chicago Classification v4.0, a manometric diagnosis of EGJOO is always considered clinically inconclusive. A conclusive clinical diagnosis requires clinically relevant symptoms together with supportive evidence of obstruction on timed barium esophagram (TBE; preferably with a barium tablet) and/or functional lumen imaging probe (FLIP). The initial barium study in this patient was not performed as a standardized TBE, and no barium tablet was administered during the examination; FLIP was not performed. Therefore, the supportive evidence required for a conclusive clinical diagnosis was unavailable, and the HREM findings are described as showing a manometric pattern consistent with EGJOO [3]. The initial esophagogastroduodenoscopy (EGD) performed at an outside hospital in October 2024 was interpreted at the time as showing no definite mucosal abnormality at the cardia (**C**), and the endoscope passed through the EGJ without significant resistance. EGD is the initial procedure of choice for excluding pseudoachalasia. The underlying malignancy may present endoscopically with features such as focal mucosal disruption, irregular ulceration, nodules, or masses, often associated with increased resistance during endoscopic passage and rigidity at the esophagogastric junction. However, none of these findings were observed during the initial EGD. After mutual discussion between the patient and the treating clinicians at the outside hospital, a joint decision was made to pursue conservative management with clinical observation and follow-up.

**Figure 2:**
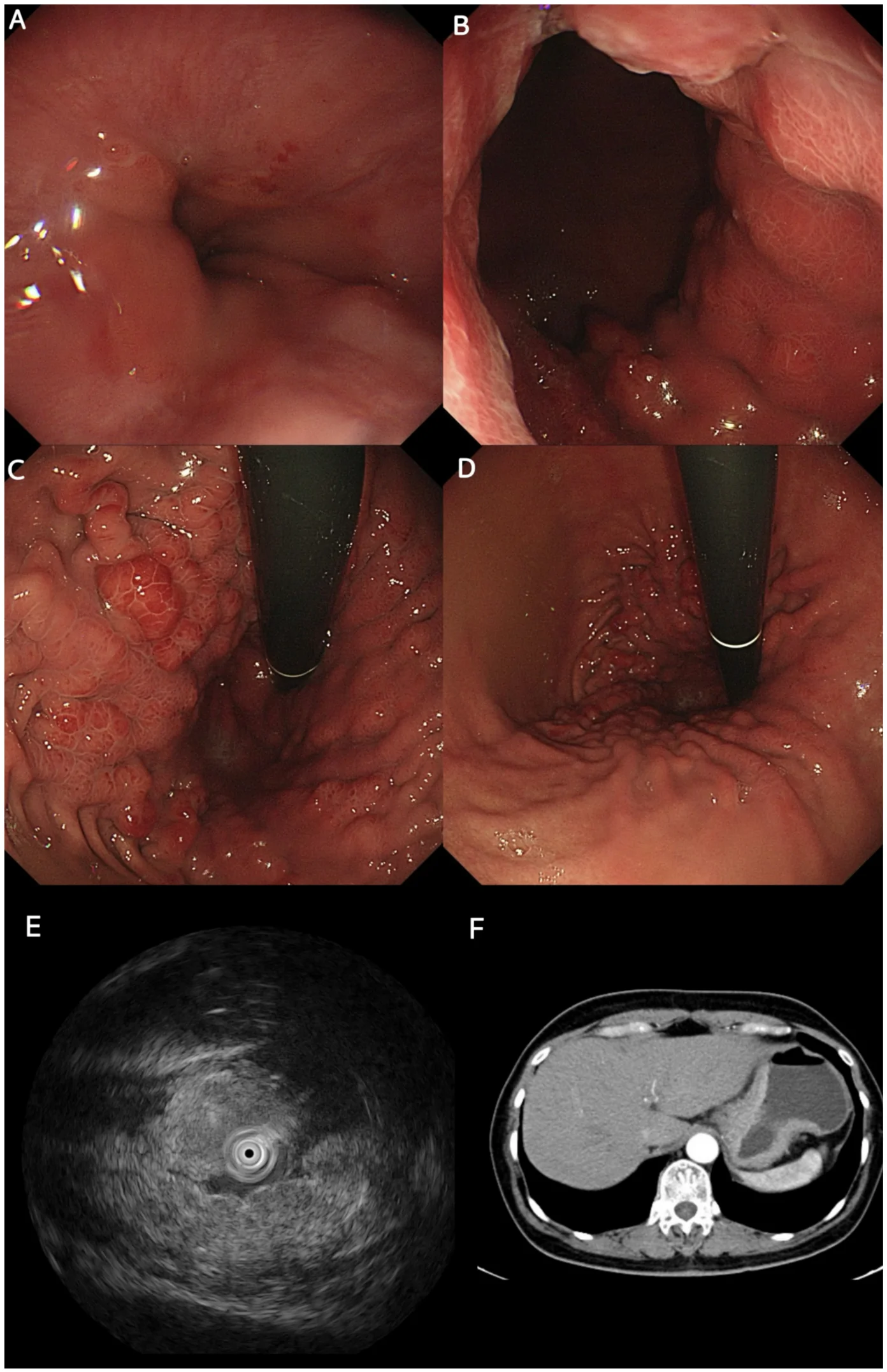
Repeat evaluation of the esophagogastric junction outflow obstruction in our hospital. The initial evaluation at an outside hospital in October 2024 included barium esophagography, HREM, and EGD. HREM was abnormal, demonstrating a manometric pattern consistent with EGJOO, whereas EGD was interpreted at the time as showing no definite mucosal abnormality. The patient did not attend scheduled follow-up and her symptoms progressively worsened between November 2024 and June 2025. She first presented to our center in July 2025, approximately nine months after the initial evaluation, when further structural evaluation was performed with repeat EGD, EUS, and contrast-enhanced CT. Repeat EGD performed at our center showed a clearly identifiable Z-line (**A**). Diffuse erythema, edema, and marked hypertrophic proliferation were noted in the cardia, gastric fundus, and upper gastric body (**B**–**D**). EUS further demonstrated marked circumferential thickening of the gastric wall, predominantly involving the deep mucosal layers (lamina propria and muscularis mucosae) and the submucosal layer (**E**). During the same examination, after EUS assessment, multiple targeted biopsies were obtained endoscopically with biopsy forceps from the abnormal mucosa on the lesser-curvature side of the gastric cardia. After the repeat EGD and EUS raised a strong suspicion of cardia cancer, a further comprehensive evaluation was undertaken. Contrast-enhanced CT was then performed and demonstrated focal thickening of the proximal gastric wall involving the fundus and lesser curvature, accompanied by prominent mucosal enhancement (**F**), raising suspicion of an infiltrative malignancy.

**Figure 3:**
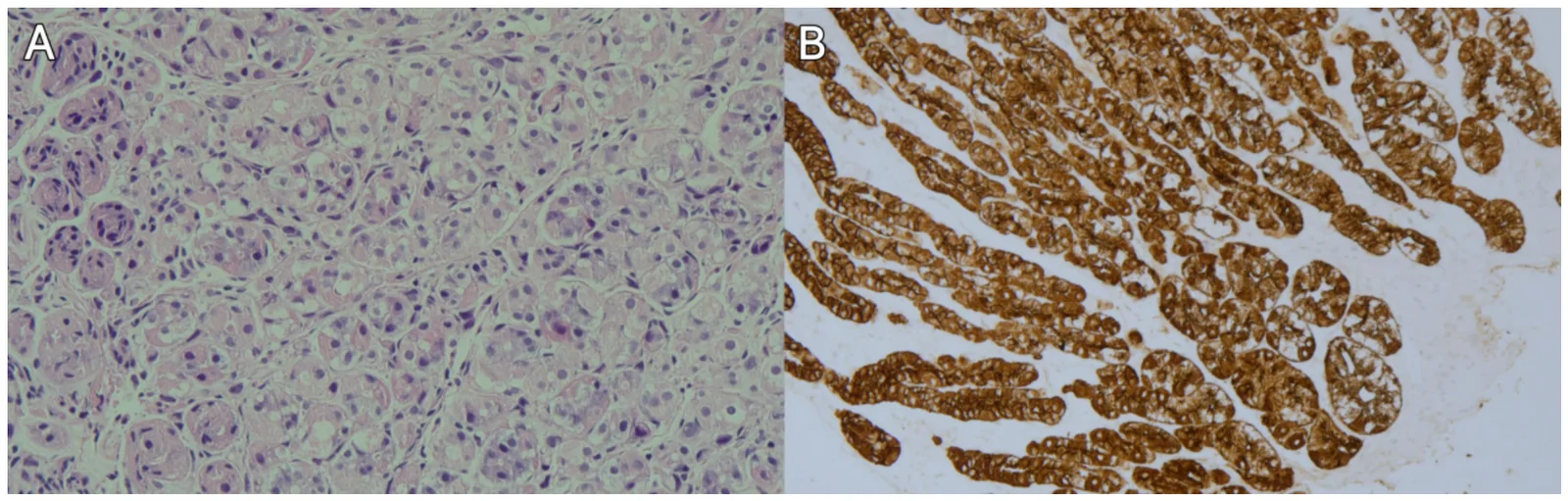
Histopathological examination of targeted biopsies obtained from the lesser-curvature side of the gastric cardia revealed poorly differentiated gastric adenocarcinoma. Histopathological examination revealed poorly differentiated gastric adenocarcinoma (**A**) (H&E ×200). Immunohistochemistry showed AE1/AE3 positivity (**B**), partial CK7 positivity, diffuse CDX-2 positivity, and a Ki-67 proliferative index of approximately 60%. The tumor was clinically staged as cT4aN1M1 (stage IV) with ovarian metastasis. The patient subsequently received chemotherapy plus cadonilimab. Further follow-up information, including treatment response and survival outcomes, was unavailable because subsequent oncologic care was provided at another institution. Pseudoachalasia is an uncommon secondary disorder that clinically mimics primary achalasia and accounts for approximately 1.5–5.4% of patients initially diagnosed with achalasia. It is most commonly caused by malignancy involving the esophagogastric junction or proximal stomach [4]. Reports have shown that tumors can cause EGJOO by infiltrating the distal esophagus or the myenteric plexus [5,6]. Early recognition is critical because delayed diagnosis may postpone potentially curative treatment. At the initial evaluation in October 2024, the patient already had progressive dysphagia, an 8 kg weight loss, a bird’s-beak appearance on barium esophagography, and a manometric pattern consistent with EGJOO. The initial EGD was interpreted at the time as showing no definite mucosal abnormality, and the endoscope passed through the EGJ without significant resistance; these findings were considered reassuring, and further evaluation for a secondary cause was not pursued. In retrospect, however, the combination of progressive dysphagia, substantial weight loss, a bird’s-beak appearance, and an EGJOO manometric pattern constituted important warning features of secondary obstruction or pseudoachalasia despite the initially non-diagnostic EGD. EGJOO is a manometric pattern that does not establish its underlying etiology. Therefore, when EGJOO is accompanied by alarm features, additional evaluation for secondary structural causes should be considered. Importantly, retrospective magnified review of Figure 1C reveals subtle mucosal changes that were not recognized as abnormal at the initial EGD. However, because approximately nine months had elapsed between the initial and follow-up examinations and no histologic confirmation was available from the initial examination, the significance of these retrospective findings remains uncertain, and they cannot establish that malignancy was present at that time. In addition, the initial retroflexed view was obtained at some distance from the cardia, potentially limiting visualization of subtle lesions. These observations underscore the importance of careful, close retroflexed inspection of the gastric cardia. In addition, EUS offers additional information regarding wall thickening and facilitates targeted biopsy, playing a crucial role in evaluating organic or motility disorders [7]. Its diagnostic utility is well established when conventional endoscopy findings are non-diagnostic, yet clinical suspicion of pseudoachalasia persists [8]. This case underscores that in patients with persistent or progressive symptoms despite an initial non-diagnostic EGD, repeat endoscopy, CT, and EUS may facilitate the detection of an underlying structural cause [9].

## Data Availability

The raw data supporting the conclusions of this article will be made available by the authors on request.
